# Supplementary material for: RNA polymerase clamp conformational dynamics: long-lived states and modulation by crowding, cations, and nonspecific DNA binding
Source: Nucleic Acids Res. 2021 Feb 15;49(5):2790–802. doi: 10.1093/nar/gkab074 (PMC7969002; doi:10.1093/nar/gkab074)
Supplement: gkab074_Supplemental_File [file gkab074_supplemental_file.pdf]

Supplementary Figures

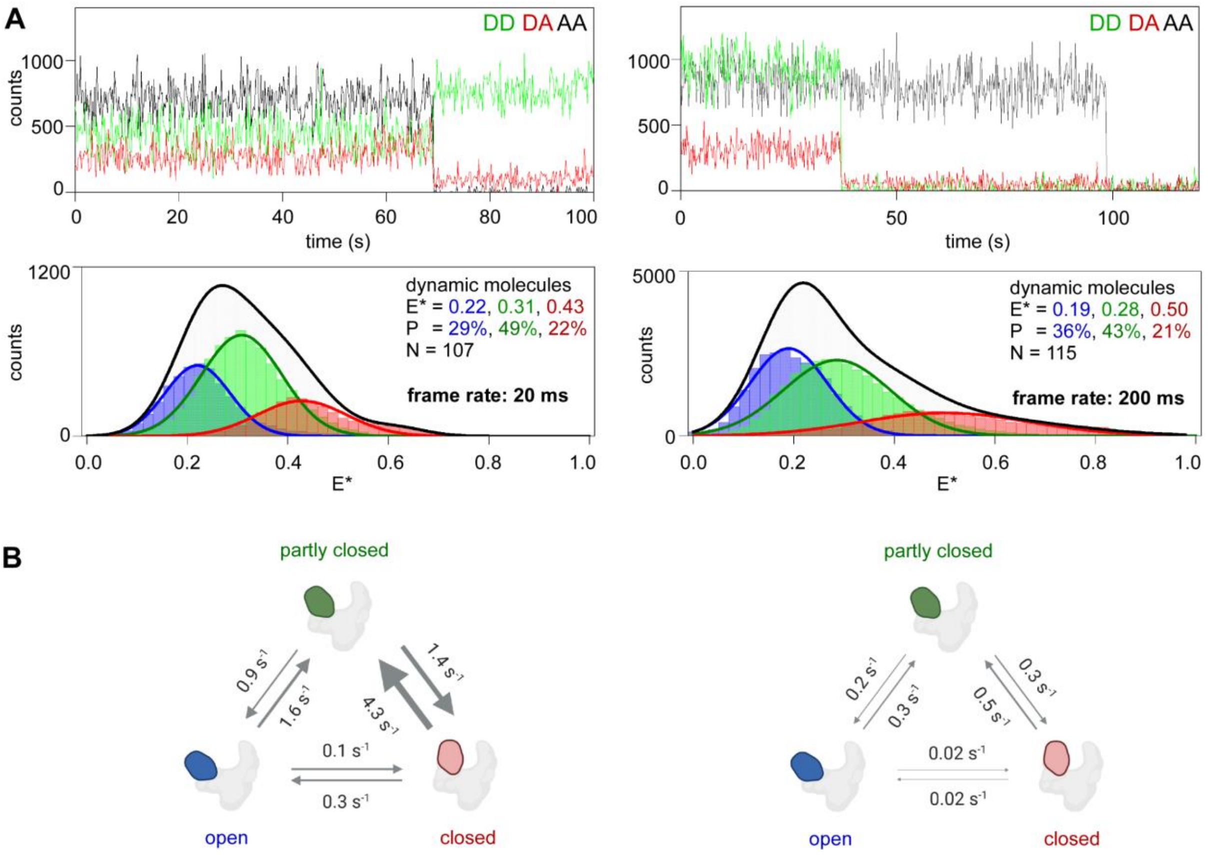

Figure S1: A. (top panels) Background corrected intensity vs time-trajectories of donor emission upon donor excitation (DD; green), acceptor emission upon donor excitation (DA; red) and acceptor emission upon acceptor excitation (black; AA); Frame rate is 200-ms; (bottom panels) Histograms and Gaussian fits of HMM-derived E* distributions from experiments with 20-ms (*left*) and 200-ms (*right*) frame rate for dynamic molecules showing open (blue), partly closed (green) and closed (red) clamp states; P, subpopulation percentage; N, number of molecules. **B.** Transition rates between open, partly closed and closed conformations obtained from experiments performed at a frame rate of 20-ms (*left*) and 200-ms (*right*). Blue, open clamp; green, partly closed clamp; red, closed clamp; grey, rest of RNAP.

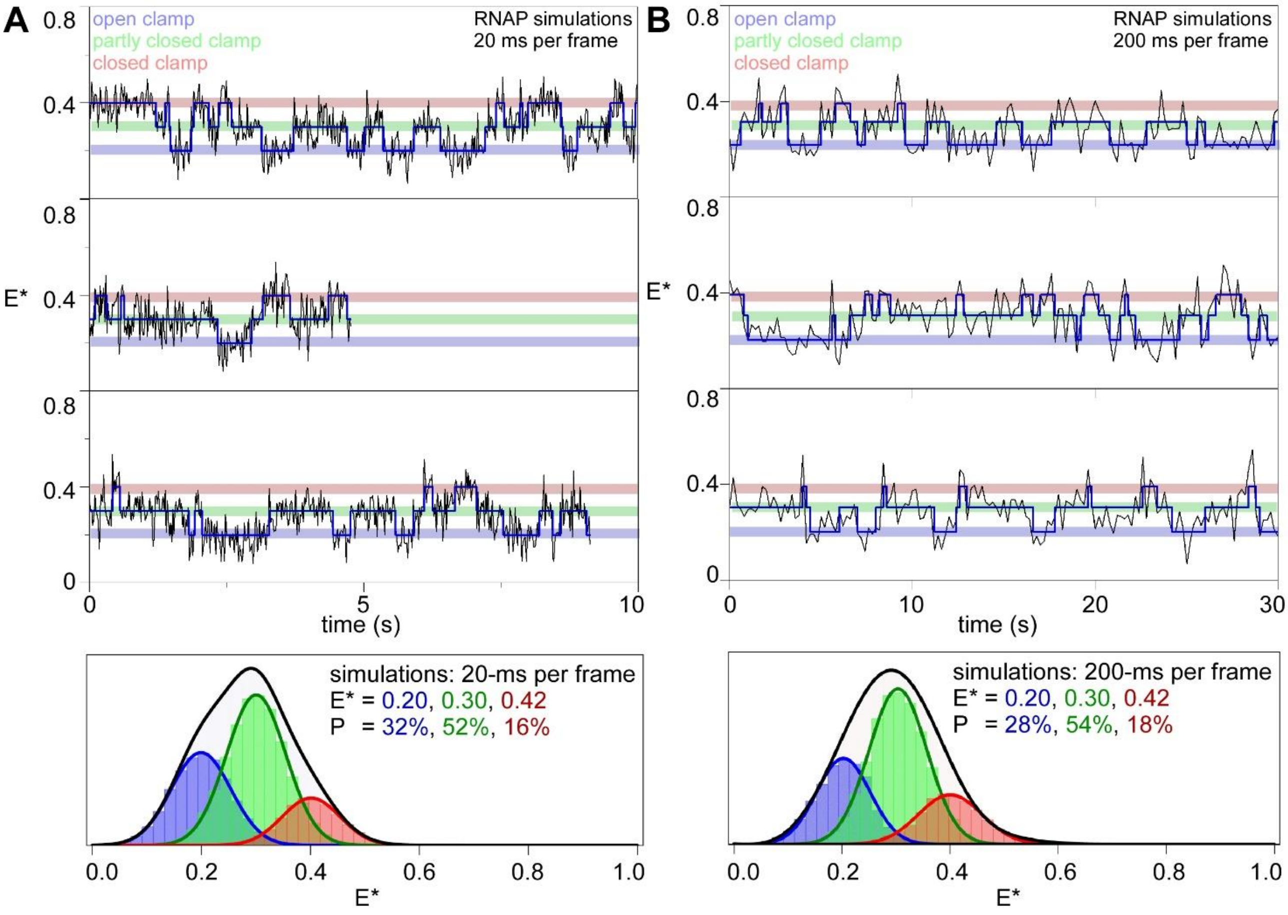

C

	τ (ms)	transition rates (s^{-1})						dwell times (s)		
		$k_{O\text{-}PC}$	$k_{O\text{-}C}$	$k_{PC\text{-}O}$	$k_{PC\text{-}C}$	$k_{C\text{-}O}$	$k_{C\text{-}PC}$	t_O	t_{PC}	t_C
RNAP	20	1.49±0.03	0.18±0.01	0.95±0.05	1.37±0.02	0.21±0.01	4.27±0.04	0.62±0.01	0.43±0.01	0.22±0.00
RNAP	200	1.16±0.01	0.15±0.00	0.56±0.00	0.71±0.00	0.33±0.00	1.30±0.00	0.80±0.00	0.80±0.00	0.60±0.00

Figure S2. A. (*top*) Representative time traces of E*, showing HMM-assigned states for simulations performed at a frame rate of 20-ms using transition rates from experiments with frame rate of 20-ms; (*bottom*) Histograms and Gaussian fits of E*, showing open (blue), partly closed (green) and closed (red) clamp states; P, subpopulation percentage. **B.** Representative time traces of E*, showing HMM-assigned states for simulations performed at a frame rate of 20-ms using transition rates from experiments with frame rate of 200-ms; (*bottom*) Histograms and Gaussian fits of E*; Colors and symbols as in A. **C**. Table showing transition rates and dwell times from simulations.

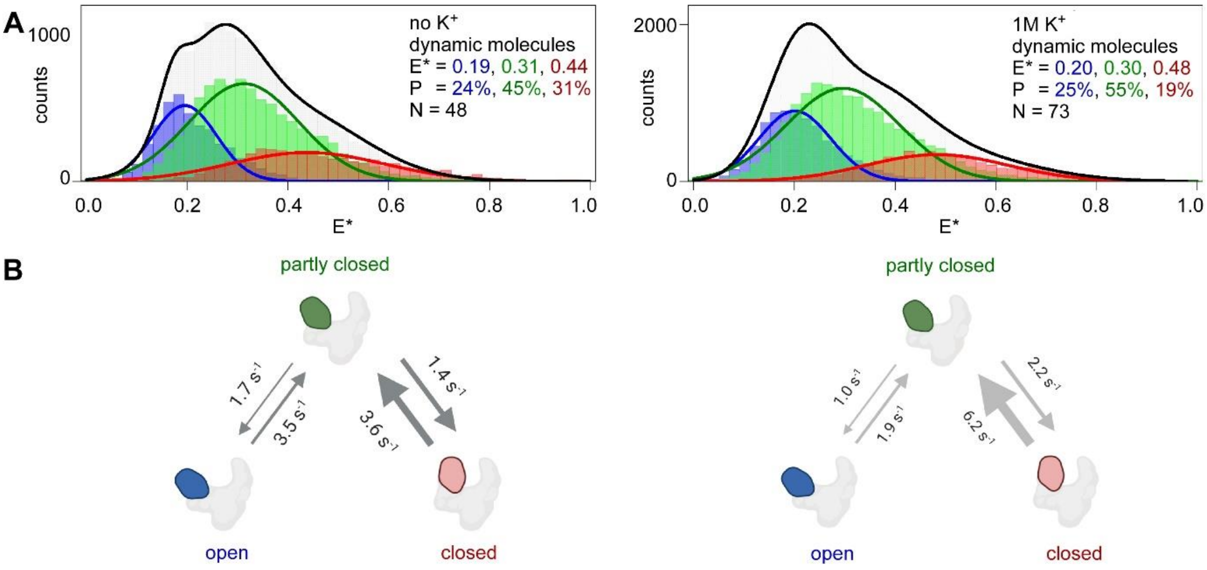

Figure S3: A. Histograms and Gaussian fits of HMM-derived E* distributions for only dynamic molecules from experiments with no K^+ (*left*) and 1000 mM K^+ (*right*) showing open (blue), partly closed (green) and closed (red) clamp states; P, subpopulation percentage; N, number of molecules. **B.** Transition rates between open, partly closed and closed conformations obtained from experiments with no K^+ (*left*) and 1000 mM K^+ (*right*). Blue, open clamp; green, partly closed clamp; red, closed clamp; grey, rest of RNAP.

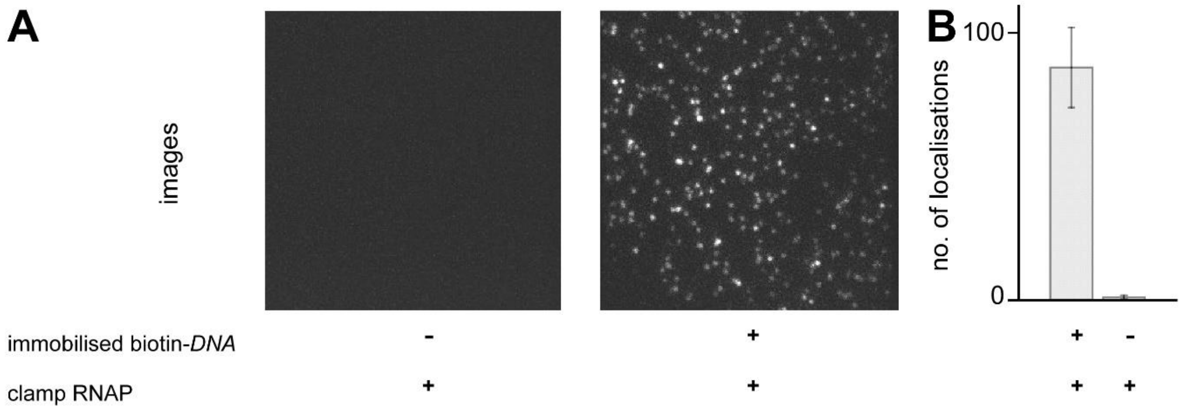

Figure S4. A. Image showing immobilisation density for clamp-labelled RNAP when added to PEG-passivated glass coverslip in absence (left) or presence (right) of immobilised non-specific DNA. **B.** Comparison of relative count for localisation of clamp-labelled RNAP.

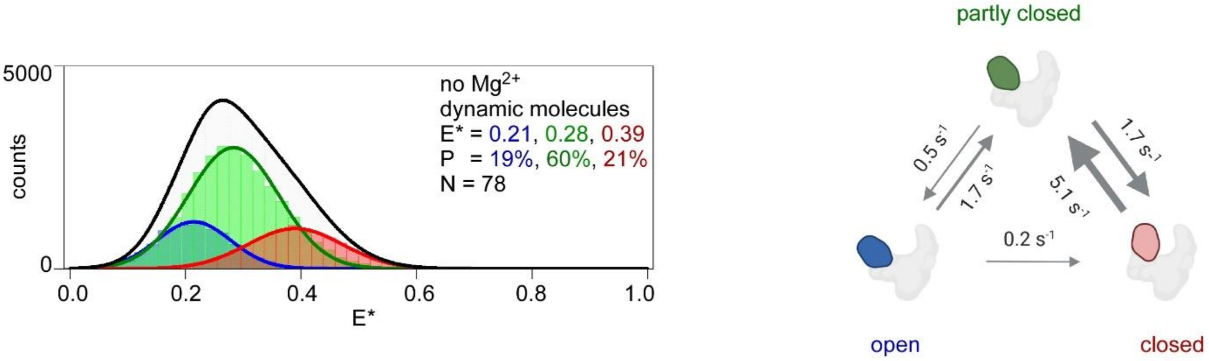

Figure S5: (*left*) Histograms and Gaussian fits of HMM-derived E* distributions for only dynamic molecules from experiments with no Mg^{2+} showing open (blue), partly closed (green) and closed (red) clamp states; P, subpopulation percentage; N, number of molecules; (*right*) Transition rates between open, partly closed and closed conformations obtained from experiments with no Mg^{2+}. Blue, open clamp; green, partly closed clamp; red, closed clamp; grey, rest of RNAP.

Table S1.

Apparent FRET efficiency (E*), width and subpopulation percentage (P) of clamp states under different conditions

	K^+ (mM)	Mg^{2+} (mM)	τ (ms)	clamp status	E*	P (%)	width	n	fig
RNAP	100	10	20	open<br>partly closed<br>closed	0.20±0.000<br>0.30±0.001<br>0.42±0.001	39.0±0.3<br>40.0±0.3<br>21.0±0.3	0.14±0.00<br>0.18±0.00<br>0.19±0.00	356	1C
RNAP	100	10	200	open<br>partly closed<br>closed	0.19±0.002<br>0.29±0.003<br>0.48±0.005	36.0±0.7<br>45.0±0.7<br>19.0±0.5	0.19±0.01<br>0.26±0.01<br>0.36±0.01	154	1D
RNAP	1000	10	20	open<br>partly closed<br>closed	0.18±0.002<br>0.29±0.003<br>0.49±0.005	43.0±0.6<br>42.0±0.6<br>15.0±0.4	0.17±0.00<br>0.25±0.00<br>0.36±0.01	216	2A
RNAP	0	10	20	open<br>partly closed<br>closed	0.19±0.002<br>0.29±0.003<br>0.44±0.006	22.1±0.6<br>61.0±0.9<br>16.5±0.6	0.13±0.01<br>0.24±0.01<br>0.36±0.01	80	2A
RNAP	100	0	20	open<br>partly closed<br>closed	0.20±0.000<br>0.29±0.001<br>0.40±0.001	38.2±0.2<br>38.3±0.2<br>24.0±0.2	0.15±0.00<br>0.18±0.00<br>0.20±0.00	266	2B
RNAP	100	25	20	open<br>partly closed<br>closed	0.22±0.001<br>-<br>0.41±0.001	68.3±0.3<br>-<br>31.7±0.3	0.19±0.00<br>-<br>0.21±0.00	288	2B
RNAP	100	100	20	open<br>partly closed<br>closed	0.20±0.001<br>-<br>0.39±0.006	91.2±0.4<br>-<br>8.8±0.4	0.19±0.00<br>-<br>0.27±0.01	244	2B
RNAP+non-specific DNA	100	10	100	open<br>partly closed<br>closed	0.20±0.001<br>0.33±0.002<br>0.44±0.001	22.9±0.2<br>24.4±0.3<br>52.7±0.3	0.18±0.00<br>0.26±0.00<br>0.24±0.00	839	4B
RNAP+20% PEG-8000	100	10	20	open<br>partly closed<br>closed	0.19±0.002<br>0.32±0.003<br>0.56±0.007	28.0±0.9<br>58.0±1.2<br>14.0±1.0	0.15±0.01<br>0.30±0.01<br>0.43±0.03	149	3B

reported errors are standard error of mean (± s.e.m).
